# Detection and characterization of an emerging type of *Babesia* sp. similar to *Babesia motasi* for the first case of human babesiosis and ticks in Korea

**DOI:** 10.1080/22221751.2019.1622997

**Published:** 2019-06-09

**Authors:** Sung-Hee Hong, Seong-Yoon Kim, Bong Goo Song, Jong-Rul Rho, Chong Rae Cho, Chul-Nam Kim, Tae-Hyun Um, Yee Gyung Kwak, Shin-Hyeong Cho, Sang-Eun Lee

**Affiliations:** a Division of Vectors and Parasitic Diseases, Korea Center for Disease Control and Prevention, Cheongju, Korea; b Department of Laboratory Medicine, Inje University Ilsan Paik Hospital, Goyang, Korea; c Department of Surgery, Inje University Ilsan Paik Hospital, Goyang, Korea; d Department of Internal Medicine, Inje University Ilsan Paik Hospital, Goyang, Korea

**Keywords:** *Babesia motasi*, *Haemaphysalis*, phylogenetic analysis, babesiosis, *Babesia microti*

## Abstract

Babesiosis is a tick-transmitted intraerythrocytic zoonosis. In Korea, the first mortalities were reported in 2005 due to Babesia sp. detection in sheep; herein we report epidemiological and genetic characteristics of a second case of babesiosis. Microscopic analysis of patient blood revealed polymorphic merozoites. To detect Babesia spp., PCR was performed using Babesia specific primers for β-tubulin, 18S rDNA, COB, and COX3 gene fragments. 18S rDNA analysis for Babesia sp., showed 98% homology with ovine Babesia sp. and with Babesia infections in Korea in 2005. Moreover, phylogenetic analysis of 18S rDNA, COB, and COX3 revealed close associations with *B. motasi*. For identifying the infectious agent, *Haemaphysalis longicornis* (296) and *Haemaphysalis flava* (301) were collected around the previous residence of the babesiosis patient. Babesia genes were identified in three *H. longicornis*: one sample was identified as *B. microti* and two samples were 98% homologous to *B. motasi*. Our study is the first direct confirmation of the infectious agent for human babesiosis. This case most likely resulted from tick bites from ticks near the patient house of the babesiosis patient. *H. longicornis* has been implicated as a vector of *B. microti* and other Babesia sp. infections.

## Introduction

Babesiosis, caused by intraerythrocytic protozoans of the Genus *Babesia*, is a zoonotic tick-borne disease, but also can be transmitted through blood transfusion [1,2]. Babesiosis is an emerging health concern and has been progressively diagnosed beyond known endemic areas [3–5]. More than 100 *Babesia* species cause infections in many wild and domestic animals, with varying degrees of virulence among different species of *Babesia* in humans and animals. *B. microti*, *B. divergens*, and *B. venatorum* are the three most predominant species known to infect humans, while other species, *B. ovis*, *B. major*, *B. bovis*, *B. bigemina*, *B. ovata*, *B. orientalis*, *B. motasi*, and *B. caballi* are causal agents for animal infections [6].

Four Genera, *Rhipicephalus*, *Ixodes*, *Haemaphysalis*, and *Hyalomma* within the *Ixodidae* have been reported as vectors of *Babesia* spp. Geographically, *Ixodes scapularis* in the USA, *I. Ricinus* in Europe, and *I. persulcatus* in Asia transmit *Babesia* parasites to natural hosts [7–9]. *B. ovis*, *B. motasi*, and *B. crassa* are primarily transmitted via *Haemaphysalis qinghaiensis* and *H. longicornis* [10]. In addition, *B. bovis* and *B. bigemina* are also transmitted primarily via *Rhipicephalus microplus* and *R. annulatus*. Recently, *B. divergens*, *B. microti*, and *B. venatorum* were detected in *I. persulcatus* and *B. divergens* in *H. longicornis* was detected in China.

Human cases of *B. microti*, *B. duncani*, and *B. divergens*-like infections have been reported in several regions in the USA [1,11–13]. Sporadic cases of infection by *B microti-*like or uncharacterized *Babesia* species were reported in Africa, South America, and Asia [1,14]. In East Asia, cases of human babesiosis have been reported in Japan [15] and Taiwan [16], caused by *B. microti*-like parasites; however, the patients were asymptomatic. In Korea, the first case of human babesiosis with a suspected *B. ovis-*like infection was reported in 2005; however, there were no epidemiological investigations conducted to determine the accepted vectors at that time [17].

The purpose of this study was to: (i) identify the *Babesia* sp., reported as a novel large *Babesia* parasite infecting humans; (ii) investigate the distribution and diversity of *Babesia* sp. in ticks collected around the patient’s previous residence; and (iii) analyse the phylogenetic association of the *Babesia* sp. detected in the patient with that detected in associated ticks.

## Materials and methods

### Blood staining

A blood sample was obtained from the jugular vein in the patient that presented with dizziness and general weakness. Thin blood films were made on dry and clean slides, air-dried, and fixed with methyl alcohol for 5 min, followed by staining with 5% Giemsa solution [18]. The slide was stained for 30 min and then dried for 30 min. Thereafter, the stained films were examined via light microscopy at ×100 magnification with an oil-immersion lens for blood parasites (Leica Microsystems, Heebrugg, Singapore). Percentage parasitemia was determined by counting the number of parasitized erythrocytes and dividing that by the total number of red blood cells (RBCs): % parasitemia = (infected RBCs/RBCs) × 100. A minimum of 500 RBCs were counted and RBCs infected with multiple parasites were counted as a single infected cell.

### DNA isolation from blood

Genomic DNA was extracted from blood samples, using a DNeasy Blood and Tissue Kit (Qiagen, Hilden, Germany) in accordance with the manufacturer’s instructions. The extracted DNA was eluted in 100 µL of elution buffer. The quantity and quality of the isolated DNA were assessed with a spectrophotometer Nanodrop 2000c (Thermo Fisher Scientific, Massachusetts, USA) and stored at −20°C until use.

### Tick collection and DNA extraction

Questing ticks were collected by flagging and a dry ice bait trap in Hoengseong-gun, Gangwon-do, Korea as described by H.S. Ginsberg and C.P. Ewing [19]. The collection area was divided into each three parts, adjusting the differential collecting methods depending on short and long grasses for efficient tick sampling regardless of tick spp. The ticks with short grasses were collected by flagging and those with long grasses were collected by dry ice trap. A total of 597 ticks were collected in the survey area. Genomic DNA was isolated from individual ticks, using the DNeasy tissue kit (Qiagen) in accordance with the manufacturer’s instructions. Quantity and quality of the isolated DNA were assessed with a spectrophotometer Nanodrop 2000c (Thermo Fisher Scientific) and stored at −20°C until use.

### Polymerase chain reaction (PCR) assays

*Babesia* spp. were detected via PCR for 18S rDNA, Cytochrome b (*COB*) and Cytochrome c oxidase subunit III (*COX3*) in accordance with a previously described method [20–23] (Table 1). *B. microti* and *B. divergens* were detected via PCR, as described previously [24–26]. Reactions were carried out in 20-μL reaction mixtures by using AccuPower PCR master mix (Bioneer, Daejeon, Korea) containing 1 μM each of the 1st forward and reverse primers, sterile water, and 500 ng of DNA template. Amplification products were electrophoresed using an auto-electrophoresis device (QIAxcel, Hilden, Germany) [27]. The PCR products were then purified using an agarose gel DNA purification kit (Qiagen) [27]. TA cloning was performed using the TOPO TA cloning kit with isolated PCR products for sequencing (Invitrogen, Carlsbad, CA, USA). These samples were sequenced using an ABI PRISM 3730xl Analyzer (Applied Biosystems, Foster City, CA, USA).

**Table 1.** Forward and reverse primers used for the detection of *Babesia* spp.

Gene		Primers		Amplication protocol	Size	Ref
18S rRNA (*Babesia* sp.)	1st PCR	BTH 1F BTH 1R	5′-CCTGAGAAACGGCTACCACATCT-3′ 5′-TTGCGACCATACTCCCCCCA-3′	94°C: 10 min, 45 cycles: (95°C: 30 s, 68°C: 1 min, 72°C: 1 min) 72°C: 10 min	561 bp	[20]
	2nd PCR	GF2F	5′-GTCTTGTAATTGGAATGATG-3′	94°C: 10 min, 40 cycles: (95°C: 30 s, 60°C: 1 min, 72°C: 1 min) 72°C: 10 min		
		GR2R	5′-CCAAAGACTTTGATTTCTCTC-3′			
18S rRNA (*Babesia* sp.)		EUK F EUK R	5′-ACCTGGTTGATCCTGCCAGT-3′ 5′-TGATCCTTCTGCAGGTTCACCTAC-3′	94°C: 5 min, 35 cycles:(94°C: 1 min, 55°C: 1 min, 72°C: 1 min 30s) 72°C: 10 min	∼1.7 kb	[21]
Cytochrome b (*COB*) (*Babesia sp*.)		COB F	5′-CCATAGCAATTAATCCAGCTA-3′	94°C: 10 min, 35 cycles: (94°C: 40 s, 54°C: 30 s, 72°C: 1 min) 72°C: 10 min	550 bp	[22]
		COB R	5′-ACCTTGGTCATGGTATTCTGG-3′			
Cytochrome c oxidase subunit III (*COX-3*) (*Babesia* sp.)		COX3 F	5′-TCAACAAAATGCCAATATGT-3′	94°C: 10 min, 35 cycles: (94°C: 40 s, 54°C: 30 s, 72°C: 1 min) 72°C: 10 min	552bp	[23]
		COX3 R	5′-AAGTGCATCTTTGGGAGAAG-3′			
*β*-tubulin (*B. microti*)	1st PCR	Tubu93 F Tubu897 R	5′-GAYAGYCCCTTRCAACTAGAAAGAGC-3′ 5′-CGRTCGAACGAACATTTGTTGHGTCARTTC-3′	95°C: 10 min, 35 cycles: (95°C: 30 s, 58°C: 1 min, 72°C: 1 min 30 s) 72°C: 10 min	551 bp	[24]
	2nd PCR	Tubu192 F Tubu782 R	5′-ACHATGGATTCTGTTAGATCYGGC-3′ 5′-GGGAADGGDATRAGATTCACAGC-3′			
18S rRNA (*B. divergens*)		B.diver F	5′-GTTTCTGMCCCATCAGCTTGAC-3′	94°C: 10 min, 45 cycles: (94°C: 30 s, 61°C: 30 s, 72°C: 1 min) 72°C: 10 min	353 bp	[25]
		B.diver R	5′-CAATATTAACACCACGCAAAAATC-3′			

### Sequence analysis

PCR products were purified and sequenced in both directions, using the specific primers. Nucleotide sequences were analysed using BLAST-N and aligned with ClustalW. The pairwise distance was analysed using the Kimura’s 2-parameter model. Phylogenetic analyses were conducted using the software MEGA 6 [28]. The neighbor-joining method was employed to construct a phylogenetic tree. The reliability of the branches in the tree was evaluated via bootstrapping analysis with 1000 replicates [29], and a bootstrap value more than 60% was considered significant.

## Results

### Clinical signs

A 70-year-old man was hospitalized with a history of dizziness and general weakness for 2 d. The patient received medication for diabetes mellitus, hypertension, and hyperlipidaemia, and underwent total pancreatectomy for intraductal papillary mucinous neoplasm 19 months ago. The patient resided in a suburban area in Hoengseong-gun, Gangwon-do, Korea. Initial analyses of vital signs were: a blood pressure 86/30 mmHg; pulse rate, 138 beats/min; respiratory rate, 20 breaths/min; body temperature, 39.5°C. Initial laboratory analysis: leukocyte count, 8840/μL with 93.5% neutrophils; haemoglobin, 14.5 g/dL; platelet count, 90,000/μL; blood urea nitrogen, 38.1 mg/dL; creatinine, 3.03 mg/dL; total bilirubin, 2.49 mg/dL; aspartate transaminase (AST), 336 IU/L; alanine transaminase (ALT), 86 IU/L; C-reactive protein, 8.2 mg/dL. Abdominopelvic computed tomography revealed no suspicious focal infection or local and distant metastasis. Piperacillin/tazobactam were administered as an initial empirical antimicrobial therapy. Blood analysis on the second day, 22 h after hospitalization changed as follows: leukocyte count, 6590/μL with 90.7% neutrophils; haemoglobin, 11.9 g/dL; platelet count, 21,000/μL; blood urea nitrogen, 33.0 mg/dL; creatinine, 2.21 mg/dL; total bilirubin, 7.98 mg/dL; AST, 1,010 IU/L; ALT, 191 IU. The patient’s consciousness was drowned and the oxygen saturation decreased to 82–83%. Chest X-ray revealed no abnormal findings. The patient was transferred to the intensive care unit and subjected to intubation and continuous renal replacement therapy; however, the patient died 36 h after hospitalization. No microorganisms were isolated from the blood culture.

### Microscopic findings

**Figure 1.**
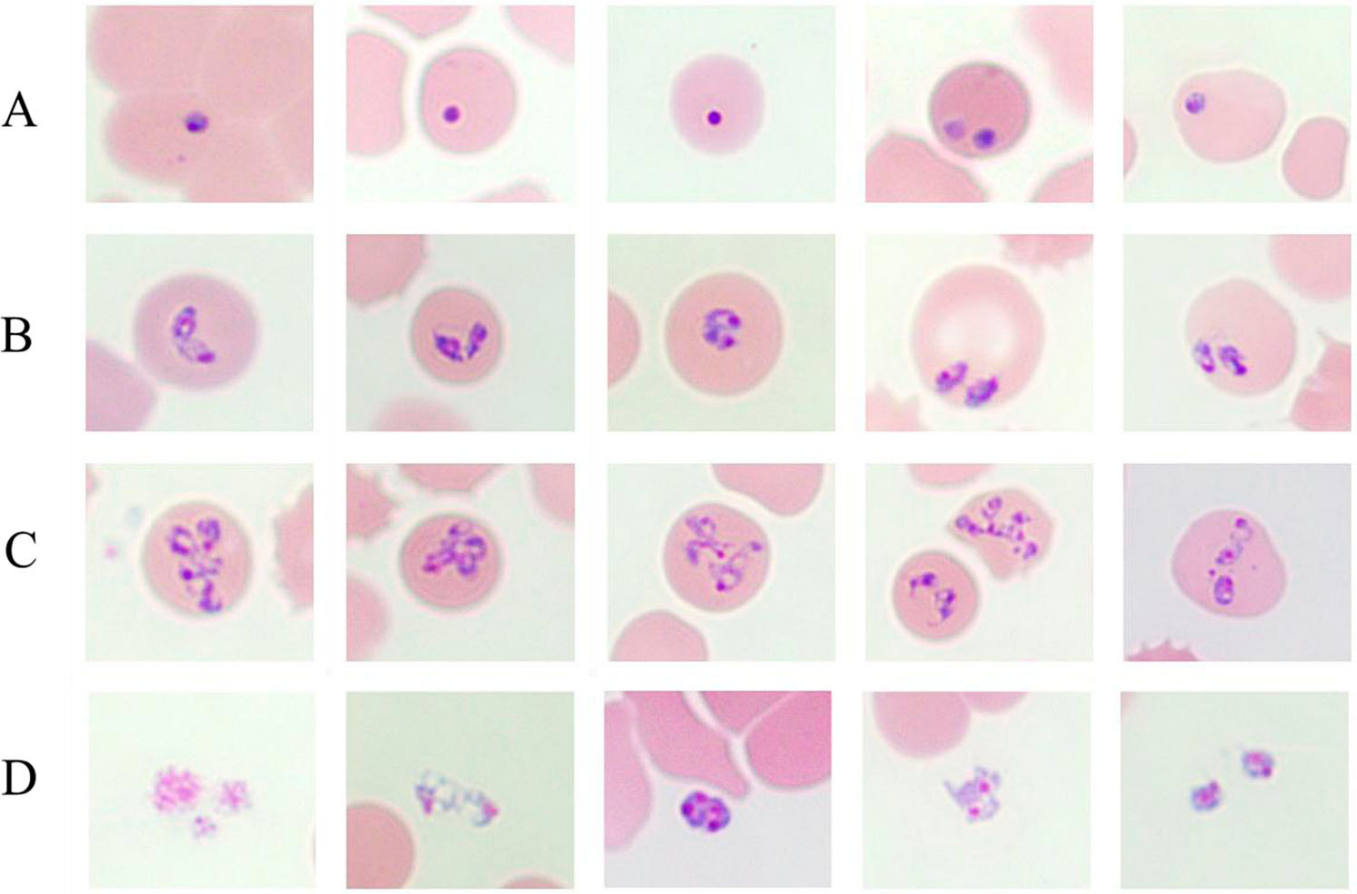
*Babesia* spp in a thin blood smear stained with 5% Giemsa on May 30, 2017, from a patient in Hoengseong-gun, Gangwon-do, Korea, showing pleomorphic and pyriform rings and multiple-infected RBCs. Pigment not present in any of the parasites. (A) Ring-form parasites; (B) Paired-pyriform parasites; (C) Pleomorphic ring forms and multiply infected parasites; (D) Cluster of extracellular rings.

Upon light microscopic examination, variable intraerythrocytic parasites as ring forms, pear-shaped forms, paired pyriforms, pleomorphic ring forms, and multiple-infected parasites and clusters of extracellular rings were detected in Giemsa-stained blood smears. The percentage of parasitaemia was 1.8% (Figure 1). Maltese cross forms comprising four masses in an erythrocyte that are often described as a characteristic of *B. microti* infection were not detected in most blood smears (Figure 1).

### Molecular characteristics of *Babesia* sp. in human

**Figure 2.**
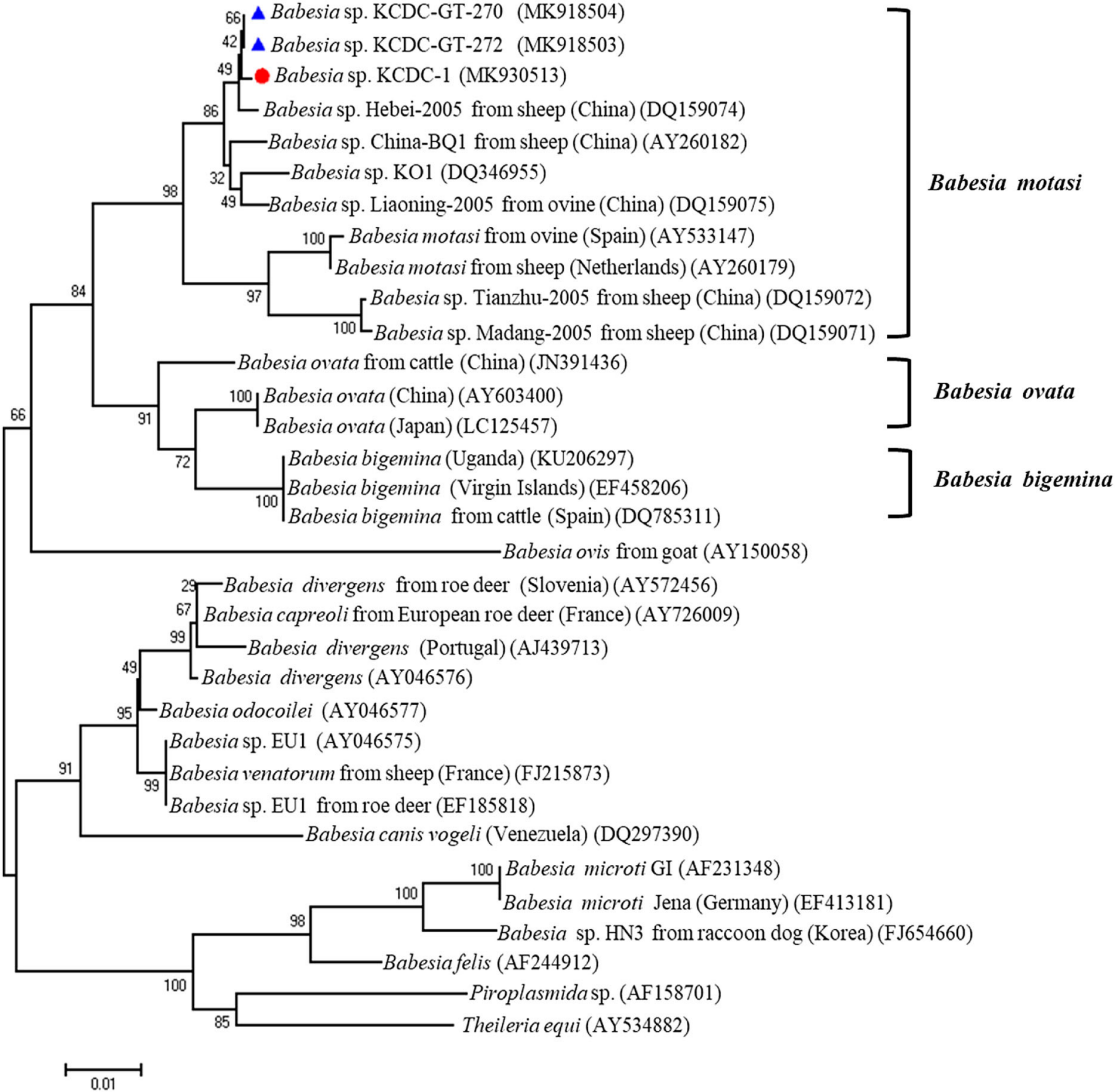
Phylogenetic relationships based on the 18S rRNA gene sequence of *Babesia* species in a human babesiosis sample and in ticks, *Babesia* sp. KCDC-GT-270, *Babesia* sp. KCDC-GR-272 and *Babesia* sp. KCDC-1, in accordance with the polymerase chain reaction-amplified sequence. The evolutionary history was inferred via the Neighbor-Joining method. The percentage of replicate trees wherein the associated taxa clustered together in the bootstrap test (1000 replicates) are shown next to the branches. Evolutionary analyses were conducted using MEGA6 (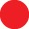
*Babesia* positive in this study) (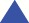
*Babesia* positives in ticks).

**Figure 3.**
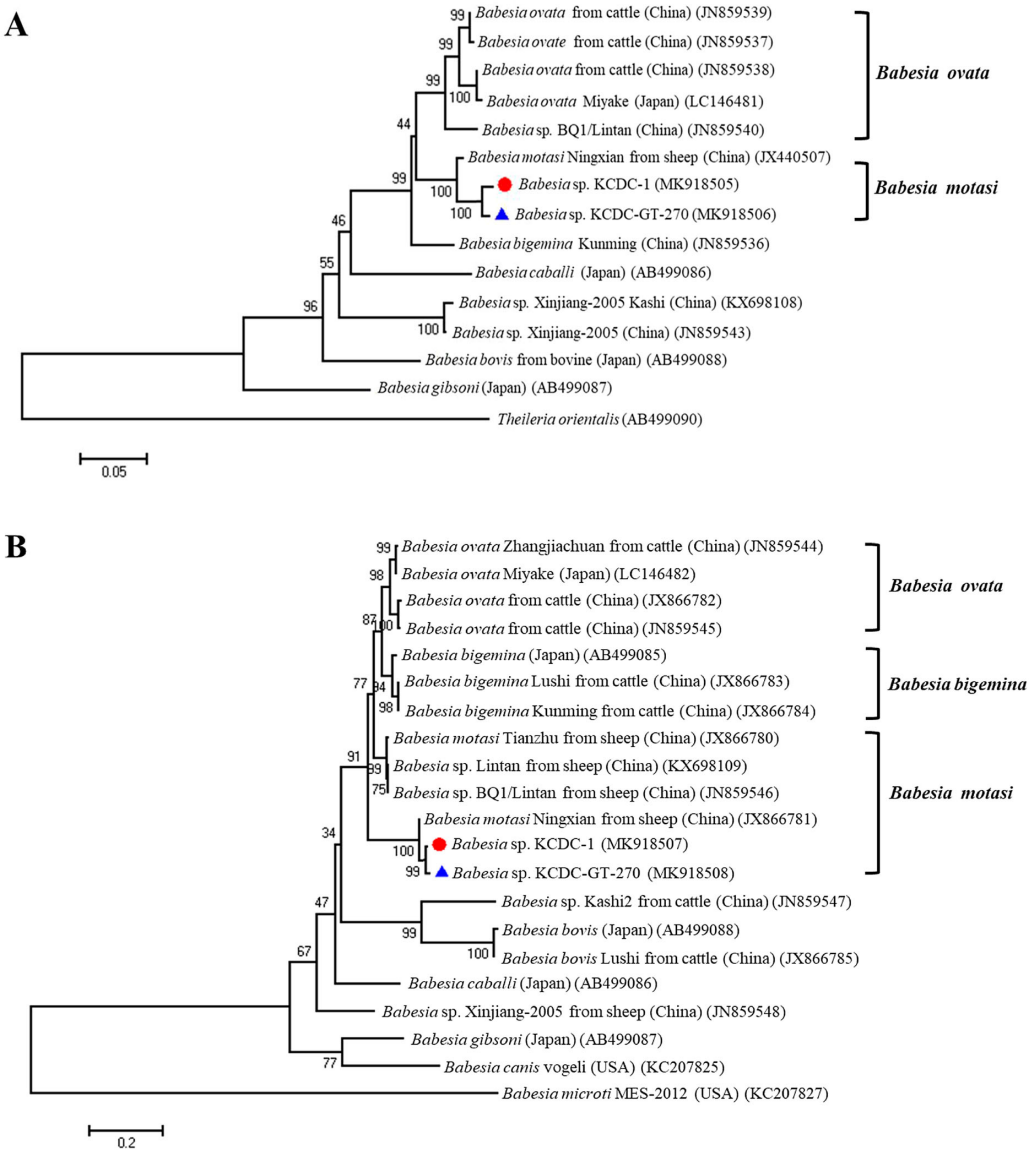
Phylogenetic relationships based on *COB* (A) and *COX3* (B) sequence of *Babesia* species in a human babesiosis sample and ticks*, Babesia* sp. KCDC-GT-270 and *Babesia* sp. KCDC-1, in accordance with the polymerase chain reaction-amplified sequence. The evolutionary history was inferred via the Neighbor-Joining method. The percentage of replicate trees wherein the associated taxa clustered together in the bootstrap test (1000 replicates) are shown next to the branches. Evolutionary analyses were conducted using MEGA6 (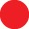
*Babesia* positive in this study) (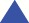
*Babesia* positives in ticks).

Genomic DNA was extracted from the patient’s blood sample and subjected to 18S rDNA analysis for *Babesia* species, Beta-tubulin for *B. microti*, and 18S rDNA for *B. divergens*. Only the 18S rDNA, *Babesia* sp. KCDC-1 (MK930513) was positive for *Babesia* species. Consequently, the samples were subjected to full-length (∼1.7 kb) amplification of 18S rDNA for *Babesia* spp. Samples with positive results upon PCR sequenced sequences then subjected to phylogenetic analysis with related *Babesia* species for 18S rRNA gene sequences in GenBank. The analysis demonstrated that the positive sequences were closely related to *Babesia* sp. KO1, ovine *Babesia* sp. Liaoning 2005, and ovine *Babesia* sp. Hebei-2005 (DQ346955, DQ159075, and DQ159074) (Figure 2). To further evaluate the classification of each sample, the *Babesia* species was subjected to genomic DNA analysis using *COB* and *COX3* gene fragments of *Babesia* species. Positive sequences from the analysis of both genes were identified and sequenced. The sequences were then phylogenetically analysed with *COB*, *Babesia* sp. KCDC-1 (MK918505) and *COX3*, *Babesia* sp. KCDC-1 (MK918507) sequences of related *Babesia* species in GenBank. This analysis demonstrated that the positive sequences of *COB* and *COX3* were closely related with ovine *Babesia motasi* Ningxian (JX440507) (Figure 3(A)) and ovine *B. motasi* Ningxian (JX866781) (Figure 3(B)), respectively. As these sequences were clustered with *B. motasi* in the phylogenetic tree, the parasite detected from the patient’s blood belonged to ovine *B. motasi*.

### Identification and molecular detection of *Babesia* sp. in questing ticks

**Figure 4.**
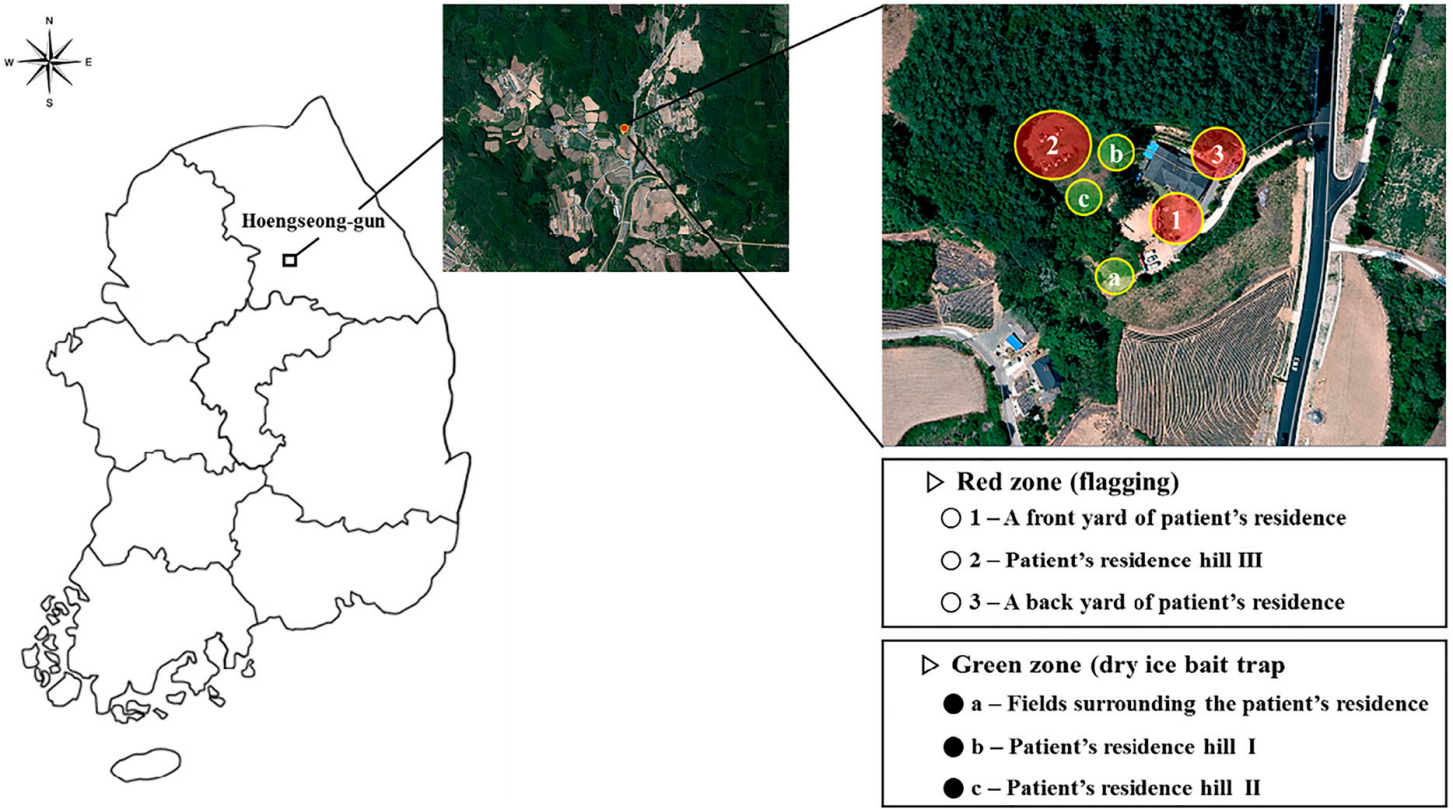
Location of the sampling site and collection methods surrounding the *Babesia*-positive patient’s residence in Hoengseong-gun, Gangwon-do, Korea.

Tick collections were performed by dividing the area around the patient’s residence into six parts (1: front yard of patient’s residence, 2: patient’s residence hill III, 3: back yard of patient’s residence, a: Fields surrounding the patient’s residence, b: patient’s residence hill I, c: patient’s residence hill II) (Figure 4). A total of 597 ticks were collected around the patient’s residence, including 296 *H. longicornis* (186 adult, 41 nymphs, and 68 larvae) and 301 *Haemaphysalis flava* (1 adult and 300 larvae) (Table 2). Among these, 94% of the ticks were collected in both the front yard of patient’s residence (442 ticks) and associated hill III (124 ticks). Based on the results of the amplification of *Babesia* genes in each tick, 2 (0.3%) were positive for 18S rDNA of *Babesia* species, 1 (0.2%) for *COB* and *COX3*, and 1 (0.2%) for *β*-tubulin gene of *B. microti*. While the nymph of *H. longicornis* yielded a positive result for only 18S rDNA, one female tick of *H. longicornis* yielded positive results for 18S rDNA, *COB*, and *COX3* gene fragments. Also, one female tick of *H. longicornis* only yielded positive results for *β*-tubulin gene of *B. microti* (Table 3).

**Table 2.** Species, stage, gender, and number of collected ticks in *Babesia-*positive patient’s residence.

Collecting site	*Haemaphysalis longicornis*				*Haemaphysalis flava*		Total No. (%)
	Female	Male	Nymph	Larva	Male	Larva	
A front yard of patient’s residence	148	1	26	30	–	237	442 (74.0)
A back yard of patient’s residence	7	–	2	–	–	–	9 (1.5)
Patient residence hill III	18	–	6	37	–	63	124 (20.8)
Fields surrounding the patient’s residence	3	–	–	–	–	–	3 (0.5)
Patient residence hill I	5	–	4	–	1	–	10 (1.7)
Patient residence hill II	5	–	3	1	–	–	9 (1.5)
No	186	1	41	68	1	300	597

**Table 3.** Detection of *Babesia* DNA in the collected ticks via PCR.

Genus/species	Gender/stage	No.	Number (%) of ticks containing DNA of			
			*Babesia* spp (18S rRNA)	*Babesia* spp (*CoB*/*Cox-3*)	*B. microti* (*β-*tubulin)	*B. divergens* (18S rRNA)
*Haemaphysalis longicornis*	Adult	187	1 (0.5)	1 (0.5)	1 (0.5)	–
	Nymph	41	1 (2.4)	–	–	–
	Larva	68	–	–	–	–
*Haemaphysalis flava*	Adult	1	–	–	–	–
	Larva	300	–	–	–	–
No.		597	2 (0.3)	1 (0.2)	1 (0.2)	0

### Molecular characteristics of *Babesia* sp. in questing ticks

**Figure 5.**
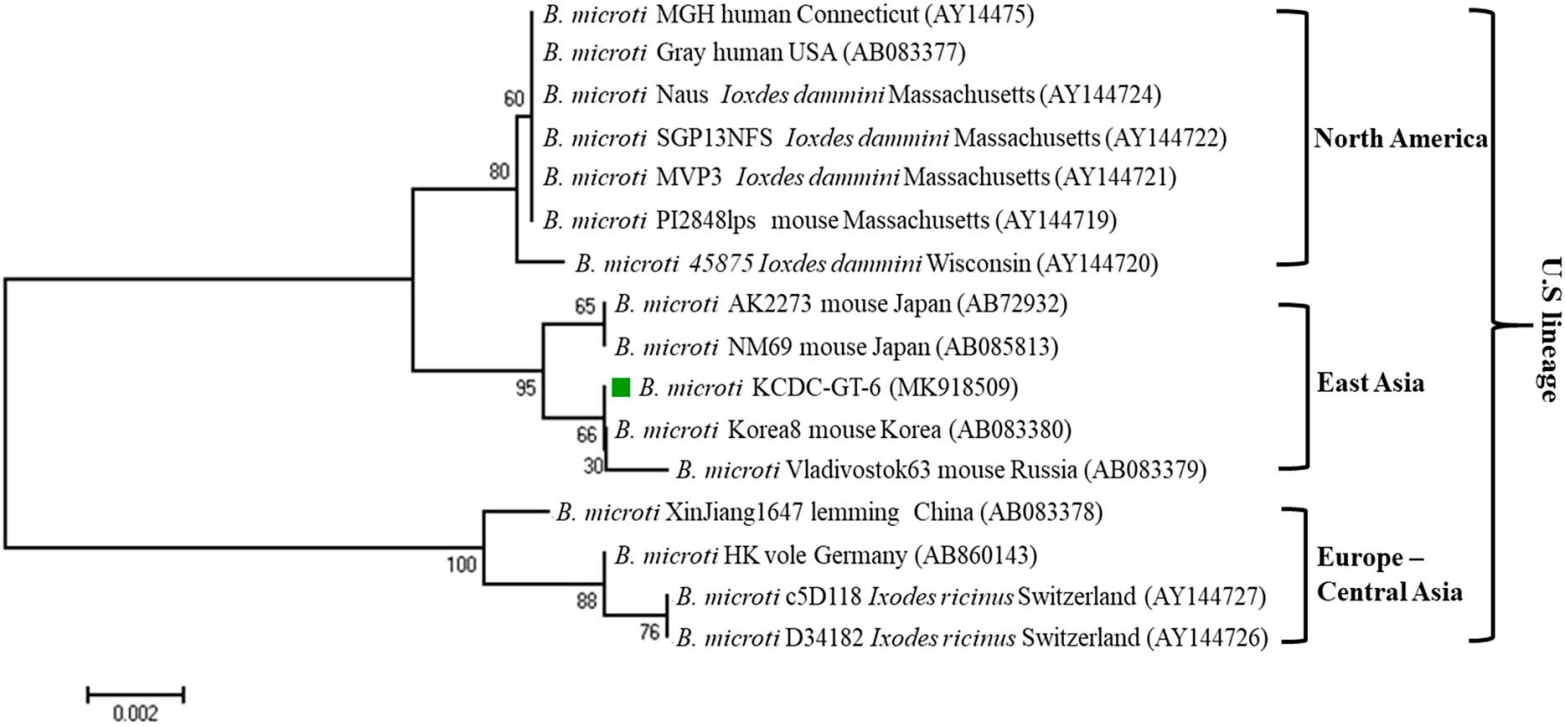
Phylogenetic relationships based on the *β*-tubulin gene sequence of *Babesia microti* in a tick, *B. microti* KCDC-GT-6, in accordance with the polymerase chain reaction-amplified sequence. The evolutionary history was inferred via the Neighbor-Joining method. The percentage of replicate trees wherein the associated taxa clustered together in the bootstrap test (1000 replicates) are shown next to the branches. Evolutionary analyses were conducted using MEGA6 (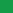
*B. microti* positive in ticks).

Sequencing of the PCR product allowed for the determination of *Babesia* species. The sequences are then phylogenetically analysed with related *Babesia* species for 18S rDNA, *COB*, *COX3*, and *β*-tubulin sequences in GenBank. Sequences of the 18S rRNA gene, *Babesia* sp. KCDC-GT-270 and KCDC-GT-272 (MK918504 and MK918503) in 2 *H. longicornis* were closely related with that of ovine *Babesia* sp. Hebei-2005 (DQ159074) (Figure 2). Also, the sequence of *COB*, *Babesia* sp. KCDC-GT-270 (MK918506) and *COX3*, *Babesia* sp. KCDC-GT-270 (MK918508) in *H. longicornis* was very closely related with ovine *Babesia* sp. Ningxian (JX866781) (Figure 3(A,B)). Since these sequences clustered with *B. motasi*, the *Babesia* sp. detected from *H. longicornis* was identified as ovine *B. motasi*. The sequence of the *β*-tubulin gene, *B. microti* KCDC-GT-6 (MK918509) in a female *H. longicornis* was very similar to that of the US-type *B. microti* (Figure 5). Furthermore, the *β*-tubulin sequence in the positive samples in the present study was closely related to those reported previously in Korea and Russia in East Asia.

## Discussion

The severity of *Babesia* infections in humans is rapidly becoming apparent, irrespective of whether the disease is transmitted via a tick bite or secondarily transmitted via transfusion of infected blood [30–32]. Previously, seven different *Babesia* spp., *B. microti*, *B. divergens*, *B. bovis*, *B. canis*, *B. duncani*, *B. venatorium*, and a novel *Babesia* sp. similar to ovine babesias were reported to cause human babesiosis [5,17,26,32,33]. Regarding the geographical distribution of human babesiosis, *B. microti* is the primary species responsible for disease in the USA [34] while *B. divergens* is the causative agent in Europe [35,36]. In Asia, human cases of a *B*. *microti* infections have been reported in Taiwan, Japan, China, and Mongolia [16,37,38]. In Korea, the first case of human babesiosis (KO1) was reported in 2007 and KO1 was highly related to Chinese ovine *Babesia* sp. [17]. Unfortunately, there was no epidemiological surveys conducted involving patients to determine the causative infects. Human babesiosis (KCDC-1) in 2017 was the second case identified in Korea and the sequence of *Babesia* sp. was very closely related to that of KO1 and Liaoning, China. These large *Babesia* are clearly distinct from other agents of human babesiosis based on their shape and phylogeny. These results suggest that the causative agent in their case of babesiosis is a novel large *Babesia* parasite infecting humans and may be highly fatal.

Human babesiosis infections and virulence depend on a number of factors, such as parasite species, patient, age, and host immune competency [39]. In general, old age and reduced cellular immunity are associated with a higher risk of symptomatic infection and more severe illness. In particular, most cases of severe babesiosis were reported among individuals lacking a spleen [14,40]. When *Babesia* sp. sporozoites first infect humans, they immediately target the host RBCs. Further, infected RBCs remain circulating in peripheral blood and frequently penetrate the host’s spleen [41–43]. The spleen with its lympho-reticular filter function is essential in resisting primary infections of *Babesia* sp. by eliminating infected cells from circulation. The spleen and cellular immune response play an essential role in resisting both primary and challenge infections of *Babesia* species [26]. However, the spleen is not critical for the development of immunity and its primary function is to eliminate infected cells from circulation and to stimulate phagocytosis of infected cells [36,44]. In this study, the patient had a splenectomy and red blood cells were considered to be infected with *Babesia* sp. and were not eliminated, thus resulting in the fatality, which is highly suggestive that splenectomy is one of the critical risk factors for *Babesia* sp. symptomatic infections and potential fatality.

Inside RBCs, *Babesia* sp. begins a cycle of maturation and growth. In the initial stages of their life cycle, *Babesia* spp. are morphologically indistinguishable from *Plasmodium* sp., both appearing as ring-like parasites. Duplication occurs via budding, where one ring divides into two rings. That is commonly referred to as the “figure eight” form. Budding may recur, giving rise to the tetrad form known as a Maltese Cross [42]. Both these morphological forms are unique to *Babesia* spp. and form the basis of a microscopy-based conclusive diagnosis. Based on morphology, *Babesia* spp. are divided into the small babesias including *B. gibsoni*, *B. microti*, and *B. divergens* and large babesias including *B. bovis*, *B. caballi*, and *B. canis*. While small *Babesia* spp. are 1.0–2.5-µm-long and regularly appear in the ring form and Maltese cross form, large *Babesia* spp. are 2.5–5-µm-long and regularly appears as a unique paired pyriform [26,45]. The form of parasites detected in the patient’s blood showed pleomorphic and paired pyriform rings. Hence, the *Babesia* sp. KCDC-1 was classified as large *Babesia* spp. on the basis of appearance and size.

Regarding the molecular characteristics of the parasite obtained from the patient’s blood, we performed nested PCR for 18S rDNA and the *β*-tubulin gene of *B. microti*, which has been regarded as the most probable cause of human babesiosis and has been detected in wild mice and other mammals in Korea [46,47]. When we sequenced full-length sequence of 18S rDNA of *Babesia* sp. [37,38], the sequence was identical to that of *Babesia* sp. KO1 (DQ346955) in Korea. Phylogenetic analysis, *Babesia* sp. KCDC-1 in our study formed a clade including several ovine *Babesia* spp. reported previously in China [48]. Based on the analysis of 18S rDNA amplification from the collected ticks, two sequences of *Babesia* spp. KCDC-GT-270 and KCDCGT-272 were identical to that of *Babesia* sp. KO1 (DQ346955) and *Babesia* sp. Hebei-2005 (DQ159074). Previous studies confirmed that the occurrence of additional viable sites in *COB* and *COX3* allowed for an improved interspecies differentiation of *Babesia* species [22,23]. The phylogenetic analysis for both *COB* and *COX3* indicates that *Babesia* sp. KCDC-1 and KCDC-GT-270 were highly identical and formed a clade including ovine *B. motasi* Ningxian in China (JX440507) based on *COB* and ovine *B. motasi* Ningxian in China (JX866781) based on *COX3*. This indicated that the identified *Babesia* parasites might be *B. motasi*, and this is the first study to detect *B. motasi* in human babesiosis and *H. longicornis* in Korea.

The *B. microti* Group is a varied group of globally distributed parasites of various lineages, such as the USA, Kobe, Hobetsu, and Munich [49,50]. The major lineage of the *B. microti* Group, the US lineage, includes *B. microti sensu stricto*, a causal agent of human babesiosis in the northeastern and upper midwestern US, where most human cases worldwide have been reported [9]. While pathogenicity may vary among parasite populations, the *B. microti* US lineage parasites are infectious to humans with or without a splenectomy. Recently, the US lineage parasites have been established to genetically vary in their *β*-tubulin gene sequences [51]. Moreover, parasites of the US lineage are phylogenetically clustered into 3 different sub-lineages, North America, Europe-Central Asia, and East Asia, each of which reflects the geographic origin of the parasites [9]. Previous reports from Korea have shown that *B. microti* detected in small mammals and wild animals closely aligned with the US strain [46,47,51]. Similarly, in this study, the *B. microti* detected in *H. longicornis* were closely related with the US linage and clustered into East Asia with Korea8 from a mouse in Korea and Vladivostok63 from mice in Russia. This study is the first to detect *B. microti*, of the US lineage in *H. longicornis* in Korea.

*Babesia* parasites are transmitted by the bite of ticks that have distinct geographical distributions [52]. *Ixodes* spp. ticks are common in the USA and Europe [53,54]. The primary vector of *B*. *microti* is *I. scapularis*. Other vectors of various strains of *B*. *micorti* include *I. spinipalpis*, *I. angustus*, *I. muris*, and *I. ricinus.* Furthermore, the vector of *B. divergens* and *B. venatorum* are *I. ricinus* and *I. persulcatu*, respectively [14]. In Asia, tick-borne pathogens were detected in both *Haemaphysalis* and *Ixodes* ticks [16,55–57]. In particular, *H. longicornis* is the primary tick species in China and essentially serves as a vector for several pathogens that cause anaplasmosis, babesiosis, and rickettsiosis [58]. In Korea, *H. longicornis* is the most commonly tick species infected with *Babesia*, collected from grass and vegetation [59,60]. The present results indicate that most of the collected ticks around the patient’s residence were *H. longicornis* and *H. flava*. Correspondingly, we confirmed that two of the *H. longicornis* were infected with *B. motasi* and the one was infected with *B. microti*. Recent reports indicate that *H. longicornis* is the potential carrier for *B. microti* to the vertebrate host and is assumed to serve as a tick vector of *B. motasi*-like isolates [10]. The present results suggest that *H. longicornis* ticks may serve as the predominant vector of *B. motasi* and *B. microti* in Gangwon-do, Korea.

## References

[1] Vannier E, Krause PJ. Human babesiosis. N Engl J Med. 2012;366:2397–2407. 22716978 10.1056/NEJMra1202018

[2] Herwaldt BL, Linden JV, Bosserman E, et al. Transfusion-associated babesiosis in the United States: a description of cases. Ann Intern Med. 2011;155:509–519. 21893613 10.7326/0003-4819-155-8-201110180-00362

[3] Jones KE, Patel NG, Levy MA, et al. Global trends in emerging infectious diseases. Nature. 2008;451:990–993. 18288193 10.1038/nature06536 PMC5960580

[4] Centers for Disease Control and Prevention Babesiosis surveillance – 18 states, 2011. Morb Mortal Wkly Rep. 2012;61:505–509. 22785341

[5] Schnittger L, Rodriguez AE, Florin-Christensen M, et al. *Babesia*: a world emerging. Infect Genet Evol. 2012;12:1788–1809. 22871652 10.1016/j.meegid.2012.07.004

[6] Fang LQ, Liu K, Li X-L, et al. Emerging tick-borne infections in mainland China: an increasing public health threat. Lancet Infect Dis. 2015;15:1467–1479. 26453241 10.1016/S1473-3099(15)00177-2 PMC4870934

[7] Brasseur P, Gorenflot A. Human babesial infections in Europe. Rocz Akad Med Bialymst. 1996;41:117–122. 8673796

[8] Sun Y, Liu G, Yang L, et al. *Babesia microti*-like rodent parasites isolated from *Ixodes persulcatus* (Acari: Ixodidae) in Heilongjiang Province, China. Vet Parasitol. 2008;156:333–339. 18718720 10.1016/j.vetpar.2008.05.026

[9] Zamoto-Niikura A, Morikawa S, Hanaki KI, et al. *Ixodes persulcatus* ticks as vectors for the *Babesia microti* U.S. lineage in Japan. Appl Environ Microbiol. 2016;82:6624–6632. 27590815 10.1128/AEM.02373-16 PMC5086556

[10] Niu Q, Liu Z, Yang J, et al. Genetic diversity and molecular characterization of *Babesia motasi*-like in small ruminants and ixodid ticks from China. Infect Genet Evol. 2016;41:8–15. 26976477 10.1016/j.meegid.2016.03.007

[11] Persing DH, Herwaldt BL, Glaser C, et al. Infection with a babesia-like organism in northern California. N Engl J Med. 1995;332:298–303. 7816065 10.1056/NEJM199502023320504

[12] Beattie JF, Michelson ML, Holman PJ. Acute babesiosis caused by *Babesia divergens* in a resident of Kentucky. N Engl J Med. 2002;347:697–698. 12200568 10.1056/NEJM200208293470921

[13] Sonenshine DE, Michael RR. Biology of ticks. 2nd ed New York (NY): Oxford University Press; 2014.

[14] Hunfeld KP, Hildebrandt A, Gray JS. Babesiosis: recent insights into an ancient disease. Int J Parasitol. 2008;38:1219–1237. 18440005 10.1016/j.ijpara.2008.03.001

[15] Saito-Ito A, Tsuji M, Wei Q, et al. Transfusion-acquired, autochthonous human babesiosis in Japan: isolation of *Babesia microti*-like parasites with hu-RBC-SCID mice. J Clin Microbiol. 2000;38:4511–4516. 11101588 10.1128/jcm.38.12.4511-4516.2000 PMC87629

[16] Shih CM, Liu LP, Chung WC, et al. Human babesiosis in Taiwan: asymptomatic infection with a *Babesia microti*-like organism in a Taiwanese woman. J Clin Microbiol. 1997;35:450–454. 9003614 10.1128/jcm.35.2.450-454.1997 PMC229598

[17] Kim JY, Cho S-H, Joo H-N, et al. First case of human babesiosis in Korea: detection and characterization of a novel type of *Babesia* sp. (KO1) similar to ovine babesia. J Clin Microbiol. 2007;45:2084–2087. 17392446 10.1128/JCM.01334-06 PMC1933034

[18] Saal JR. Giemsa *stain* for the diagnosis of bovine babesiosis. I. Staining properties of commercial samples and their component dyes. J Protozool. 1964;11:573–582. 14231188 10.1111/j.1550-7408.1964.tb01801.x

[19] Ginsberg HS, Ewing CP. Comparison of flagging, walking, trapping, and collecting from hosts as sampling methods for northern deer ticks, *Ixodes dammini*, and lone-star ticks, *Amblyomma americanum*. Exp Appl Acarol. 1989;7:313–322. 2806016 10.1007/BF01197925

[20] Zintl A, Finnerty EJ, Murphy TM, et al. Babesias of red deer (*Cervus elaphus*) in Ireland. Vet Res. 2011;42:7. 21314977 10.1186/1297-9716-42-7 PMC3037898

[21] Medlin L, Elwood HJ, Stickel S, et al. The characterization of enzymatically amplified eukaryotic 16S-like rRNA-coding regions. Gene. 1988;71:491–499. 3224833 10.1016/0378-1119(88)90066-2

[22] Tian Z, Luo J, Zheng J, et al. Phylogenetic analysis of *Babesia* species in China based on Cytochrome b (COB) gene. Infect Genet Evol. 2013;13:36–40. 23041715 10.1016/j.meegid.2012.09.001

[23] Tian Z, Liu G, Yin H, et al. Cytochrome c oxidase subunit III (COX3) gene, an informative marker for phylogenetic analysis and differentiation of *Babesia* species in China. Infect Genet Evol. 2013;18:13–17. 23619098 10.1016/j.meegid.2013.04.002

[24] Zamoto A, Tsuji M, Kawabuchi T, et al. US-type *Babesia microti* isolated from small wild mammals in Eastern Hokkaido, Japan. J Vet Med Sci. 2004;66:919–926. 15353841 10.1292/jvms.66.919

[25] Tavassoli M, Tabatabaei M, Mohammadi M, et al. PCR-based detection of *Babesia* spp. infection in collected ticks from cattle in West and North-West of Iran. J Arthropod Borne Dis. 2013;7:132–138. 24409438 PMC3875879

[26] Homer MJ, Aguilar-Delfin I, Telford SR 3rd, et al. Babesiosis. Clin Microbiol Rev. 2000;13:451–469. 10885987 10.1128/cmr.13.3.451-469.2000 PMC88943

[27] Talameh J, Misher A, Hoskins J. A capillary electrophoresis method for genotyping the 9-bp exon 1 insertion/deletion in BDKRB2. Pharmacogenomics. 2012;13:353–358. 22304584 10.2217/pgs.11.171 PMC3290903

[28] Kumar S, Nei M, Dudley J, et al. MEGA: a biologist-centric software for evolutionary analysis of DNA and protein sequences. Brief Bioinform. 2008;9:299–306. 18417537 10.1093/bib/bbn017 PMC2562624

[29] Felsenstein J. Confidence limits on phylogenies: an approach using the bootstrap. Evolution. 1985;39:783–791. 28561359 10.1111/j.1558-5646.1985.tb00420.x

[30] Leiby DA. Transfusion-transmitted *Babesia spp*.: bull’s-eye on *Babesia microti*. Clin Microbiol Rev. 2011;24:14–28. 21233506 10.1128/CMR.00022-10 PMC3021205

[31] Leiby DA. Transfusion-associated babesiosis: shouldn’t we be ticked off? Ann Intern Med. 2011;155:556–557. 21893616 10.7326/0003-4819-155-8-201110180-00363

[32] Gubernot DM, Nakhasi HL, Mied PA, et al. Transfusion-transmitted babesiosis in the United States: summary of a workshop. Transfusion. 2009;49:2759–2771. 19821952 10.1111/j.1537-2995.2009.02429.x

[33] Calvo de Mora A, Garcia Castellano JM, Herrera C, et al. Human babesiosis: report of a case with fatal outcome. Med Clin. 1985;85:515–516. 4079511

[34] Vannier E, Krause PJ. Update on babesiosis. Interdiscip Perspect Infect Dis. 2009;2009:984568. 19727410 10.1155/2009/984568 PMC2734943

[35] Centeno-Lima S, do Rosario V, Parreira R, et al. A fatal case of human babesiosis in Portugal: molecular and phylogenetic analysis. Trop Med Int Health. 2003;8:760–764. 12869099 10.1046/j.1365-3156.2003.01074.x

[36] de Vos AJ, Dalgliesh RJ, Callow LL. Babesia. In: Soulsby EJL, editor. Immune responses in parasitic infections. Boca Raton, FL: CRC Press; 1987; Vol. III, Protozoa; p. 183–222.

[37] Wei Q, Tsuji M, Zamoto A, et al. Human babesiosis in Japan: isolation of *Babesia microti*-like parasites from an asymptomatic transfusion donor and from a rodent from an area where babesiosis is endemic. J Clin Microbiol. 2001;39:2178–2183. 11376054 10.1128/JCM.39.6.2178-2183.2001 PMC88108

[38] Yao LN, Wei R, Zeng CY, et al. Pathogen identification and clinical diagnosis for one case infected with *Babesia*. Zhongguo Ji Sheng Chong Xue Yu Ji Sheng Chong Bing Za Zhi. 2012;30:118–121. 22908812

[39] Ord RL, Lobo CA. Human babesiosis: pathogens, prevalence, diagnosis and treatment. Curr Clin Microbiol Rep. 2015;2:173–181. 26594611 10.1007/s40588-015-0025-z PMC4649939

[40] Gorenflot A, Moubri K, Precigout E, et al. Human babesiosis. Ann Trop Med Parasitol. 1998;92:489–501. 9683900 10.1080/00034989859465

[41] Cursino-Santos JR, Halverson G, Rodriguez M, et al. Identification of binding domains on red blood cell glycophorins for *Babesia divergens*. Transfusion. 2014;54:982–989. 23944874 10.1111/trf.12388 PMC3880634

[42] Lobo CA, Rodriguez M, Cursino-Santos JR. *Babesia* and red cell invasion. Curr Opin Hematol. 2012;19:170–175. 22488304 10.1097/MOH.0b013e328352245a

[43] Lobo CA. *Babesia divergens* and *Plasmodium falciparum* use common receptors, glycophorins A and B, to invade the human red blood cell. Infect Immun. 2005;73:649–651. 15618210 10.1128/IAI.73.1.649-651.2005 PMC538995

[44] Khan N, editor. Emerging protozoan pathogens. London: Taylor and Francis; 2008; p. 303–349.

[45] Ramgopal Laha MD, Sen A. Morphology, epidemiology, and phylogeny of *Babesia*: an overview. Trop Parasitol. 2015;5:94–100. 26629451 10.4103/2229-5070.162490 PMC4557164

[46] Hong SH, Kim H-J, Jeong Y-I, et al. Serological and molecular detection of *Toxoplasma gondii* and *Babesia microti* in the blood of rescued wild animals in Gangwon-do (Province), Korea. Korean J Parasitol. 2017;55:207–212. 28506045 10.3347/kjp.2017.55.2.207 PMC5450965

[47] Hong SH, Lee S-E, Jeong Y-I, et al. Prevalence and molecular characterizations of *Toxoplasma gondii* and *Babesia microt*i from small mammals captured in Gyeonggi and Gangwon Provinces, Republic of Korea. Vet Parasitol. 2014;205:512–517. 25178555 10.1016/j.vetpar.2014.07.032

[48] Liu AH, Yin H, Guan GQ, et al. At least two genetically distinct large *Babesia* species infective to sheep and goats in China. Vet Parasitol. 2007;147:246–251. 17531391 10.1016/j.vetpar.2007.03.032

[49] Goethert HK, Telford SR. What is *Babesia microti*? Parasitology. 2003;127:301–309. 14636016 10.1017/s0031182003003822

[50] Tsuji M, Wei Q, Zamoto A, et al. Human babesiosis in Japan: epizootiologic survey of rodent reservoir and isolation of new type of *Babesia microti*-like parasite. J Clin Microbiol. 2001;39:4316–4322. 11724838 10.1128/JCM.39.12.4316-4322.2001 PMC88542

[51] Zamoto A, Tsuji M, Wei Q, et al. Epizootiologic survey for *Babesia microti* among small wild mammals in northeastern Eurasia and a geographic diversity in the beta-tubulin gene sequences. J Vet Med Sci. 2004;66:785–792. 15297749 10.1292/jvms.66.785

[52] Spielman A, Wilson ML, Levine JF, et al. Ecology of *Ixodes dammini*-borne human babesiosis and Lyme disease. Annu Rev Entomol. 1985;30:439–460. 3882050 10.1146/annurev.en.30.010185.002255

[53] Spielman A. Human babesiosis on Nantucket Island: transmission by nymphal *Ixodes* ticks. Am J Trop Med Hyg. 1976;25:784–787. 1008124 10.4269/ajtmh.1976.25.784

[54] Walter G. Transmission and course of parasitemia of *Babesia microti* (Hannover I strain) in the bank vole (*Clethrionomys glareolus*) and field vole (*Microtus agrestis*). Acta Trop. 1984;41:259–264. 6150621

[55] Uilenberg G. International collaborative research: significance of tick-borne hemoparasitic diseases to world animal health. Vet Parasitol. 1995;57:19–41. 7597784 10.1016/0304-4017(94)03107-8

[56] Fujisaki K. Development of acquired resistance precipitating antibody in rabbits experimentally infested with females of *Haemaphysalis longicornis* (Ixodoidea: Ixodidae). Natl Inst Anim Health Q (Tokyo). 1978;18:27–38. 77478

[57] Ica A, Vatansever Z, Yildirim A, et al. Detection of *Theileria* and *Babesia* species in ticks collected from cattle. Vet Parasitol. 2007;148:156–160. 17614205 10.1016/j.vetpar.2007.06.003

[58] Yongshuai P, Qi M, Jian F, et al. Molecular identification of tick-borne pathogens in tick *Haemaphysalis longicornis* from sheep in Henan, China. Turk J Vet Anim Sci. 2017;41:51–55.

[59] Kim CM, Yi Y-H, Yu D-H, et al. Tick-borne rickettsial pathogens in ticks and small mammals in Korea. Appl Environ Microbiol. 2006;72:5766–5776. 16957192 10.1128/AEM.00431-06 PMC1563606

[60] Kang SW, Doan HTT, Choe SE, et al. Molecular investigation of tick-borne pathogens in ticks from grazing cattle in Korea. Parasitol Int. 2013;62:276–282. 23501057 10.1016/j.parint.2013.02.002

